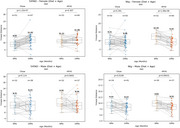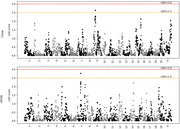# Investigating Hyperactivity in Alzheimer's Disease: Genetic and Dietary Influences in AD‐BXD Mice

**DOI:** 10.1002/alz70861_108412

**Published:** 2025-12-23

**Authors:** Kiyoung Kim, Sungjoon Park, Soorin Yim, Dongyun Kim, Doyeong Hwang, Kyungwook Lee, Daniel M Gatti, Amy R Dunn, Elissa J Chesler, Kristen MS O'Connell, Soonyoung Lee

**Affiliations:** ^1^ LG AI Research, Gangseo‐gu, Seoul Korea, Republic of (South); ^2^ The Jackson Laboratory, Bar Harbor, ME USA

## Abstract

**Background:**

Alzheimer’s disease (AD) often features behavioral changes such as hyperactivity, but the genetic and environmental drivers of this phenotype remain unclear. In this study we compare 5xFAD transgenic mice—harboring five familial AD mutations that drive amyloid pathology—with non‐transgenic (Ntg) littermates, which lack these mutations and serve as controls. This direct 5xFAD vs. Ntg comparison allows us to isolate the impact of AD‐related pathology on activity levels in aging animals.

**Method:**

Hyperactivity was phenotyped in AD‐BXD mice using the 5xFAD transgenic model. Behavioral data were collected as Y‐maze walking distances at two ages (6 and 14 months) across 46 strains to assess age‐related changes in activity. Additionally, the impact of diet on hyperactivity was evaluated, with mice that were fed either normal chow or a high‐fat/high‐sugar (HFHS).

**Result:**

At 14 months of age, female 5xFAD mice showed a significant increase in walking distance compared to their 6‐month‐old counterparts, indicating a progressive hyperactivity phenotype with aging. In contrast, Ntg mice exhibited a decrease in walking distance over the same period, suggesting an age‐related decline in activity (Figure 1).

• The hyperactivity score was calculated as follows: ΔDistance_5xFAD_ ‐ ΔDistance_Ntg_

Quantitative trait locus mapping (QTL) revealed LOD ≥ 2.5 peaks on chromosome 9 under the chow diet and on chromosome 7 under the HFHS diet (Figure 2). Each locus contains multiple candidate genes previously implicated in AD or hyperactivity regulation, which will be followed up in targeted functional studies.

**Conclusion:**

This study shows that hyperactivity in AD‐BXD mice arises from an interplay of genetic and environmental factors. Diet‐specific QTLs on chromosomes 9 and 7 point to candidate genes for AD‐related hyperactivity. These loci warrant targeted functional validation and may inform novel interventions for behavioral symptoms in AD.